# Epidemiology of *Achromobacter* in a French hospital over six years: sample types, species, and antibiotic resistance profiles

**DOI:** 10.1016/j.nmni.2026.101777

**Published:** 2026-05-30

**Authors:** Léa Cultrera, Caroline Demeule, Catherine Neuwirth, Anne Claire Saussier, Véronique Varin, Lucie Amoureux, Arnaud Magallon

**Affiliations:** a Université Bourgogne Europe, CHU Dijon Bourgogne, Department of Clinical Bacteriology, Dijon, 21000, France; b UMR AgroEcologie 1347, INRAE, University of Bourgogne Europe, Dijon, France; c French Observatory on Achromobacter, Department of Clinical Bacteriology, CHU Dijon Bourgogne, 21000, France

**Keywords:** *Achromobacter*, Epidemiology, Identification, Opportunistic pathogen, Resistance

## Abstract

**Background:**

*Achromobacter* are non-fermenting-Gram-negative pathogens, mainly reported in cystic fibrosis (CF), but also responsible for opportunistic infections among immunocompromised patients or in case of healthcare-associated infections. Species identification techniques require specific methods not used in the routine diagnostic laboratories (*nrdA*-gene sequencing, MultiLocus Sequence Typing or matrix-assisted laser desorption/ionization-time-of-flight mass spectrometry using in-house databases). As a result, epidemiological data are scarce, particularly outside the context of CF.

**Methods:**

In this descriptive, retrospective monocentric study, we included all *Achromobacter* isolates, identified at species-level with accurate reference methods, collected from our 1600-beds University Hospital (Dijon, France) during the period 2018-2023, from patients with (pwCF) or without CF (pw/oCF). Data about sample types, context of detection, culture characteristics (mono or polymicrobial), species distribution and antibiotic resistance profiles were collected.

**Results:**

In total, 651 *Achromobacter* strains from 274 patients (including 36 pwCF) were isolated from 570 samples. A wide variety of samples was found and mainly polymicrobial (46% of which associated with *Pseudomonas aeruginosa*). Among pw/oCF, *Achromobacter* spp. were mainly detected in respiratory samples (38.7%). More than 12 species were identified, *A. xylosoxidans* being the most frequent both in pwCF (44.4%) and pw/oCF (60.5%). Overall, piperacillin-tazobactam was the most effective antibiotic (96.5% susceptibility). In pwCF, resistance rates were significantly higher for meropenem, cotrimoxazole and ceftazidime but lower for imipenem compared with pw/oCF. *A. xylosoxidans* isolates appeared significantly more resistant to imipenem, ceftazidime and ciprofloxacin than non*-xylosoxidans Achromobacter* species isolates.

**Conclusions:**

This study highlights the importance of accurate *Achromobacter* species identification and of CF status when elaborating treatment recommendations.

## 1 Introduction

*Achromobacter* bacteria are Gram-negative, non-fermenting bacilli considered since the 2000s as emerging opportunistic pathogens mainly reported in patients with cystic fibrosis (pwCF), in immunocompromised patients and in healthcare associated infections. They are encountered in anthropized environments, mainly in wet areas such as lake shores, surface waters, soils or in hospital or domestic sanitary facilities (sinks, washbasins, showers) [1,2]. Today, 22 species have been officially validated within the *Achromobacter* genus (https://lpsn.dsmz.de/search?word=Achromobacter, last accessed on 23 April 2026). *A. xylosoxidans* is the predominant species in clinical practice, but the species occupying second place varies from region to region and according to the population studied [1,[3], [4], [5], [6]]. Identification of the strains to the species-level requires specific methods based on housekeeping-genes sequencing (MultiLocus Sequence Typing (MLST), or *nrdA-*gene sequencing), or matrix-assisted-laser-desorption/ionization-time-of-flight mass spectrometry (MALDI-TOF-MS) with specific databases, which are not used in the routine diagnostic laboratories. Indeed, conventional routine identification methods, such as biochemical-bases techniques, MALDI-TOF-MS with commercial databases, or even 16S-rRNA gene sequencing cannot accurately distinguish between *Achromobacter* species and sometimes lead to misidentification (e.g *A. insuavis* misidentified as *A. xylosoxidans* by MALDI-TOF-MS) [4,[7], [8], [9], [10]].

In pwCF, *Achromobacter* is regularly detected in respiratory samples with varying prevalences [11]. Airway chronic colonization in pwCF is associated with an increased risk of lung transplantation, mortality and reduced respiratory capacity [3,12]. Outside the context of CF (patients without CF, pw/oCF), *Achromobacter* is encountered in various samples, mainly respiratory but also in bacteremia, ear or ophthalmic infections and may be of nosocomial or community origin [1,[13], [14], [15]]. Managing patients with a positive *Achromobacter* sample remains complex. The distinction between colonization and active infection is often difficult especially in polymicrobial samples.

This bacterial genus is naturally resistant to several antibiotics such as amoxicillin, aztreonam, cephalosporins (except ceftazidime), temocillin, macrolides, mainly due to efflux mechanisms (AxyABM and AxyXY-OprZ) [16,17]. AxyXY-OprZ confers intrinsic aminoglycoside resistance in several species including *A. xylosoxidans, A. insuavis, A. dolens*, *A. ruhlandii* which are the most frequently encountered in clinical practice [1]. Acquired resistance mechanisms, such as overexpression of efflux systems (AxyABM, AxyXY-OprZ, AxyEF-OprN, AxySUV or AinCDJ), or acquisition or overexpression of betalactamases have also been described [1,[18], [19], [20], [21], [22]]. Currently, with regard to therapeutic categorization, EUCAST only offers breakpoints for the species *A. xylosoxidans* and six antibiotics (piperacillin-tazobactam, cotrimoxazole, meropenem and, more recently, cefiderocol, imipenem, and meropenem/vaborbactam). Regarding the Clinical and Laboratory Standards Institute (CLSI), no interpretive criteria are yet available for the genus *Achromobacter* and most laboratories use the M100 non-Enterobacterales criteria.

Studies conducted outside the context of CF are scarce and most of them do not use accurate species identification methods, making it impossible to determine whether certain species are more relevant than others [5,23,24]. The aim of this study was to provide a comprehensive description of the epidemiology of *Achromobacter* (nature of clinical samples, species involved and antimicrobial resistance profiles) in patients with or without CF over a 6-year period (2018-2023) in our hospital (Dijon University Hospital, France).

## 2 Materials and methods

### 2.1 Strains used in this study

This retrospective study included all *Achromobacter* strains isolated in the bacteriology department from clinical samples taken from patients admitted to Dijon University Hospital (1600 beds) between January 1, 2018, and December 31, 2023.

For the description of the types of samples (description only for pw/oCF), we included one strain per patient and sample site per year (n = 238). For the description of the species involved, we included one strain per patient and species per year (n = 274).

For the description of resistance in pw/oCF, we included only isolates recovered from clinically relevant samples (antibiograms performed as part of the patient's care) (n = 243), excluding therefore the isolates detected during screening for multidrug resistant Gram negative bacteria (MDRGN) (patients mainly in haematology and intensive care departments). We excluded duplicate isolates (from the same patient, same species and same sample site recovered within two weeks). For the evaluation of resistance in pwCF, we included all strains, because phenotypes may vary over time (n = 239).

### 2.2 Identification of species and strain typing

The strains were identified to the species level using MALDI-TOF-MS (Bruker®) in association with our local database developed previously which allows an accurate identification of 19 *Achromobacter* species. The study presenting this database reported correct identification for 99.4% of the 167 isolates [8,25]. This database allows also identifying isolates belonging to the multiresistant Ax ST137 clone [26].

The *nrdA* gene (765 bp) was sequenced for isolates that could not be reliably identified by the local database or to confirm that the identification was correct in the case of species underrepresented in the database (*A*. *animicus, A. denitrificans, A. deleyi, A. insolitus, A. kerstersii*). Isolates were identified using the pubMLST website (http://pubmlst.org/organisms/achromobacter-spp, last accessed on 23 April, 2026) [7].

In addition, strains from pwCF were subjected to genotyping by pulsed-field gel electrophoresis (PFGE), which we have been performing routinely for these patients in our department for over 25 years [27].

### 2.3 Antibiotic susceptibility tests (AST)

AST were carried out during routine bacterial analyses using the standard agar disk diffusion method in accordance with “Comité de l'antibiogramme de la Société Française de Microbiologie” (CASFM)/EUCAST recommendations (bacterial inoculums of 0.5 Mac Farland plated on Mueller Hinton agar). The diameters were collected in our laboratory software and reinterpreted according to the selected recommendations. Several references were used for diameters interpretation (Table S1), due to the lack of recommendations for all antibiotics concerning *Achromobacter*. We used the CASFM/EUCAST2025 recommendations for *A. xylosoxidans* for meropenem, piperacillin-tazobactam and cotrimoxazole. For imipenem we used the diameters recently published for *Achromobacter* spp., which constitute future CLSI recommendations [28]. The CASFM/EUCAST 2025 recommendations for *Pseudomonas aeruginosa* were used for gentamicin, amikacin, and those of CASFM/EUCAST2019 V1 for *Pseudomonas aeruginosa* were used for ceftazidime and ciprofloxacin (before the introduction of the “susceptible at increased exposure” categorization).

### 2.4 Data collection

The following data were collected using laboratory and institutional softwares: prescribing department, type of sample, and for certain strains of interest, (i.e. from respiratory or monomicrobial samples), patient's risk factors (presence or absence of a chronic pathology, immunosuppressive treatment, indwelling catheters, mechanic ventilation, tracheotomy), and in monomicrobial cases, antibiotic therapy implemented after bacterial documentation.

A respiratory sample was considered polymicrobial when *Achromobacter* was detected with at least another pathogen. For blood cultures, the infection was considered polymicrobial when the presence of *Achromobacter* was associated with that of another bacterium in the same sample or in blood cultures collected within 24 h before or after the one positive with *Achromobacter*.

### 2.5 Statistics

Comparisons were assessed using Z-test or Fisher's exact test. In longitudinal isolates from pwCF resistance evolution was analyzed using McNemar's exact test (overall). P-value <0.05 was regarded as significant.

### 2.6 Ethics

The study protocol and data collection complied with French good practice regulations (Data Protection Act No. 78-17 of January 6, 1978) and European regulations (GDPR EU 2016/679) regarding data protection and patient information (Commitment of compliance MR004 No. 2210228 of December 3, 2018). This study was registred in Health Data Hub (25666785).

## 3 Results

A total of 651 *Achromobacter* strains from 274 patients were included in this study: 242 strains from 36 pwCF and 409 from 238 pw/oCF. These strains were collected from 570 samples (163 from pwCF and 407 from pw/oCF).

### 3.1 Patients’ characteristics

For pwCF the median age was 20.8 years [1-64] and 44.6% were men. For pw/oCF the median age was 64,9 years [0−94] and 62.2% were men. *Achromobacter* spp. were isolated from patients attending a great diversity of departments, mainly pulmonology (respiratory samples), ENT (Ear, nose and throat) department (ear samples), Intensive care units (respiratory and blood cultures), endocrinology (diabetic foot samples), oncology departments (blood cultures) and the CF Center. For pwCF, all samples were respiratory specimens with the exception of 3 blood cultures (from a single patient). Among pw/oCF in whom *Achromobacter* was identified in respiratory samples, one-third of patients had chronic obstructive pulmonary disease (COPD) and more than a quarter had cancer. A context of ventilator-associated pneumonia was observed in more than 15% of cases and approximately 10% of patients had a tracheostomy tube, often long-term due to various chronic diseases (such as myopathies, oropharyngeal cancers, or neurological diseases). Concerning blood cultures (from pw/oCF and pwCF), all patients had an intravascular catheter and 17 out 21 had cancer (Table 1). Among these patients, bacteremia was proved in 15 out 21 cases, as evidenced by positive culture results from samples obtained *via* venipuncture or from at least two different devices. In patients with a positive *Achromobacter* ear swab, otitis externa was present in half of the cases. In most of the other cases, chronic otitis media was observed, with some patients presenting with cholesteatoma and/or trans-tympanic aerators. With regard to urine samples and in accordance with the French recommendations, *Achromobacter* was considered clinically significant in the majority of patients (11 out of 18). Among patients whose bone samples presented *Achromobacter* (21/238), diabetic foot infection was the most frequently observed context (n = 16).

**Table 1:** Characteristics of the 21 patients with at least one blood culture positive for *Achromobacter*.

Patient	Species	Context/risk factor	Documented bacteremia	Positive for *Achromobacter* in KT culture	Monomicrobial infection	Associated bacteria in blood cultures∗
**BCP-1**	*A. xylosoxidans*	Diabetic, KT	Y	na	Y	-
**BCP-2**	*A. marplatensis*	Cancer, KT	Y	Y	Y	-
**BCP-3**	*A. xylosoxidans*	Post-surgery, KT	Y	na	Y	-
**BCP-4**	*A. marplatensis*	Cancer, KT	Y	N	Y	-
**BCP-5**	*A. marplatensis*	Cancer, KT	Y	N	Y	-
**BCP-6**	*A. xylosoxidans*	Cancer, KT	Y	Y	N	*Stenotrophomonas maltophilia*; *Hafnia alvei*; *Comamonas spp*; *Pseudomonas* spp. ; *Acinetobacter* spp.
**BCP-7**	*A. insuavis*	Aortical anevrism, KT	Y	N	N	*Aeromonas* spp. ; *Stenotrophomonas maltophilia*; *Pseudomonas putida*; *Pseudomonas* spp. ; *Aerococcus* spp. ; *Staphylococcus cohnii*
**BCP-8**	*A. insuavis*	Cancer, KT	Y	N	N	*Pseudomonas aeruginosa*
**BCP-9**	*A. xylosoxidans*	Cancer, KT	Y	Y	N	*Pseudomonas aeruginosa*
**BCP-10**	*A. xylosoxidans*	Cancer, KT	Y	Y	N	*Pseudomonas aeruginosa*
**BCP-11**	*A. xylosoxidans*	Cancer, KT	Y	N	N	*Escherichia coli*; *Serratia marcescens*; *Stenotrophomonas maltophilia*
**BCP-12**	*A. marplatensis*	Cancer, KT	Y	na	N	*Enterobacter kobei*; *Rhizobium radiobacter*; Coagulase-negative *Staphylococcus*; *Pseudomonas* spp.
**BCP-13**	*A. xylosoxidans*	Cystic Fibrosis, KT	Y	na	N	*Pseudomonas aeruginosa*
**BCP-14**	*A. xylosoxidans*	Cancer, KT	Y	na	N	*Stenotrophomonas maltophilia*
**BCP-15**	*A. insuavis*	Cancer, KT	Y	N	N	*Klebsiella variicola*
**BCP-16**	*A. xylosoxidans*	Cancer, KT	N	N	N	*Enterobacter* spp. ; *Escherichia coli*; *Citrobacter freundii*
**BCP-17**	*A. insuavis*	Cancer, KT	N	na	N	*Delftia acidovorans*
**BCP-18**	*A. mucicolens*	Cancer, KT	N	na	N	*Enterobacter* spp. ; *Stenotrophomonas maltophilia*
**BCP-19**	*A. mucicolens*	Cancer, KT	N	N	N	*Enterococcus faecalis*
**BCP-20**	*A. xylosoxidans*	Cancer, KT	N	N	N	*Enterococcus avium*; *Enterococcus faecalis*
**BCP-21**	*A. marplatensis*	Cancer, KT	site of collection not specified	N	N	*Staphylococcus warneri*; *Staphylococcus haemolitycus*; *Acinetobacter* spp. ; *Delftia* spp. ; *Micrococus* spp. ; *Stenotrophomonas maltophilia*

KT: medical devices including central venous catheter, arterial catheter, peripheral catheter, PICC Line (Peripherally Inserted Central Catheter) or implantable chamber.

N: No Y Yes -: absence of associated bacteria na: no KT received for analysis.

Documented bacteremia: Positive result from a blood sample collected *via* venipuncture or from at least two different implanted devices.

∗: in the same sample or in blood cultures collected within 24 h before or after sampling.

### 3.2 Distribution of species

A total of 642 of the 651 *Achromobacter* strains could be identified at species level. For 9 cases the species identification could not be obtained because MALDI-TOF-MS had failed and the isolate was not available any more for sequencing. Identification was obtained by MALDI-TOF-MS coupled with our laboratory's specific database for 609 strains (94.8%, 609/642) (including 29 identifications confirmed by *nrdA* analysis for rare species). Sequencing of the *nrdA* gene enabled the identification of 33 additional strains (32 unreliable scores (<2) with MALDI-TOF-MS and 1 misidentification by MALDI-TOF-MS (*A. denitrificans* with final identification *A. other*)).

In terms of distribution, 12 species with valid names (10 in pw/oCF and 9 in pwCF) were identified. The main species detected in patients was *A. xylosoxidans* (58% of all patients) (Table 2) regardless of the year, the category of patient and the type of sample studied (considering the most frequent). In pw/oCF*, A. xylosoxidans* was detected more frequently in respiratory samples (73.1%) compared with other types of samples (53%) (taking into account a single sample per patient), this difference was significant (p < 0.05).

**Table 2:** Number of patients with a positive *Achromobacter* sample over the period 2018 – 2023, all species combined and by species.

	*2018*		*2019*		*2020*		*2021*		*2022*		*2023*		*period* *2018-2023*		
	NCF	CF	NCF	CF	NCF	CF	NCF	CF	NCF	CF	NCF	CF	NCF	CF	Total
**All species**	**33**	**15**	**34**	**16**	**46**	**13**	**50**	**13**	**51**	**3**	**38**	**5**	**238**	**36**	**274**
***A. xylosoxidans***	24	10	22	11	27	8	25	6	29	2	26	2	**144**	**16**	**160**
***A. insuavis***	2	3	5	2	2	2	8	1	5	0	3	0	**22**	**4**	**26**
***A. mucicolens***	0	1	1	1	4	1	5	2	3	0	2	0	**15**	**5**	**20**
***A. marplatensis***	0	0	1	0	3	0	2	1	6	0	4	0	**16**	**1**	**17**
***A. insolitus***	2	0	1	0	2	0	3	0	1	1	0	2	**9**	**3**	**12**
***A. aegrifaciens***	1	0	0	0	2	0	1	1	2	0	2	0	**8**	**1**	**9**
***A. animicus***	0	1	0	1	1	1	0	0	1	0	0	1	**2**	**4**	**6**
***A. kerstersii***	0	0	1	0	2	0	1	0	0	0	0	0	**4**	**0**	**4**
***A. dolens***	0	0	0	0	1	0	1	0	1	0	0	0	**3**	**0**	**3**
***A. deleyi***	0	0	1	0	0	0	0	0	0	0	0	0	**1**	**0**	**1**
***A. denitrificans***	0	0	0	1	0	0	0	0	0	0	0	0	**0**	**1**	**1**
***A. spanius***	0	0	0	0	0	0	0	1	0	0	0	0	**0**	**1**	**1**
**Other species**	0	0	2	0	3	1	3	1	4	0	0	0	**12**	**2**	**14**

NCF = patients without cystic fibrosis.

CF = patients with Cystic fibrosis.

A same patient may appear in several columns and/or rows.

Among the 36 pwCF, only 2 carried more than one species of *Achromobacter*. Around half (19/36) patients had several *Achromobacter*-positive samples and 8 of them were considered chronically colonized (more than 3 positives samples per year during at least two years), 7 by A*. xylosoxidans* and one by *A. insuavis.* PFGE analysis pointed out that all pwCF were carriers of their own strain which persisted during the analyzed period.

MALDI-TOF-MS analysis of all the *A. xylosoxidans* spectra allowed identifying a single patient carrying the ST137 clone (pwCF already known as chronically colonised with the strain in the past).

### 3.3 Distribution of the different types of samples in patients without cystic fibrosis

*Achromobacter* were detected in various samples*,* mainly in respiratory samples (Table 3). *Achromobacter* was also isolated in blood cultures, affecting 2 to 5 patients per year (20 in total).

**Table 3:** Number of patients without cystic fibrosis with *Achromobacter*-positive sample over the 2018-2023 global period, all samples combined and by sample type.

	2018	2019	2020	2021	2022	2023	Period 2018-2023
**At least one positive sample for Achromobacter**	**33**	**34**	**46**	**50**	**51**	**38**	238
**Respiratory samples**	13	14	18	22	25	12	93 (39.1%)
**Ear samples**	8	4	7	6	9	7	40 (16.8%)
**Screening for MDRGN**	3	1	5	7	4	6	25 (10.5%)
**Blood culture**	2	4	2	5	4	3	20 (8.4%)
**Diabetic foot sampling**	2	6	5	4	2	1	20 (8.4%)
**Urine samples**	4	3	3	3	2	3	18 (7.6%)
**Catheter/*Implantable catheter ports***	1	1	1	3	0	1	7 (2.9%)
**Contact lens samples**	0	1	3	0	1	1	6 (2.5%)
**Bone samples∗**	1	1	0	0	2	1	5 (2.1%)
**Vaginal samples**	0	0	2	0	0	0	2 (0.8%)
**Peritoneal fluid**	0	0	0	1	1	0	2 (0.8%)
**Maxillary sinus**	0	0	0	0	0	2	2 (0.8%)
**Other samples**	0	0	3	3	1	2	9 (3.8%)

“Other samples” included: graft preservation solution, cavum biopsy, cervical cellulitis, mastoid cavity, ankle eschar, popliteal fossa haematoma, eye, osteonecrosis pus and soft tissue of the thigh.

∗: excluding diabetic foot bone samples.

MDRGN: multidrug resistant Gram negative bacteria, on rectal or oral samples.

Respiratory samples” included sputum samples, tracheal secretions, fibro-aspirations bronchioalveolar-lavage fluids and lung biopsy.

A same patient may appear in several columns and/or rows.

### 3.4 Mono or polymicrobial nature of sample

Excluding the samples of screening for MDRGN (n = 47), 98 of the 523 positive samples were monomicrobial (19%) and 425 were polymicrobial (81%). Among the 425 polymicrobial samples, 194 were also positive with *Pseudomonas aeruginosa* (46%), (39% and 49% from pwCF or pw/oCF respectively). With regard to respiratory samples from pw/oCF, another pathogen was detected in 78% of cases in association with *Achromobacter* (such as Enterobacteriaceae, non-fermenting Gram-negative bacilli, or *Staphylococcus aureus*). Bacteremia was documented in 15 out of 21 patients representing all monomicrobial infections (5/5) as well as 10 out of 16 of polymicrobial infections (Table 1).

We focused on the patients from whom *Achromobacter* was the only pathogen isolated. It concerned 47 patients including 3 pwCF, the main sample types being respiratory samples (32 patients) followed by blood cultures, urine, ear and very few other types of samples (catheter, graft preservation solution, peritoneal fluid). Seven species were detected in this context*, A. xylosoxidans* being the main species (35/47; 74%). The most common risk factor was cancer (18/47). Other risk factors outside CF were also identified, such as immunosuppression, surgery context, diabetes, COPD, or the presence of a catheter or mechanical ventilation. Some *Achromobacter* infections were also encountered in immunocompetent patients with no identified risk factors and concerned ear samples. Overall, the antibiotics used for treatment after microbiological documentation were meropenem, ceftazidime, cotrimoxazole and piperacillin-tazobactam, the latter being the most frequently administered.

### 3.5 Epidemiology of antimicrobial resistance

Excluding the isolates detected during screening for MDRGN, the most effective antibiotics were piperacillin-tazobactam, imipenem, and meropenem while ciprofloxacin showed the poorest activity (Table 4).

**Table 4:** Proportion of susceptible strains over the period 2018 – 2023.

Antibiotic	All strains n = 482	NCF strains n = 243	CF strains n = 239	CF vs NCF p-value	First AB CF^a^ n = 39^d^	First AB CF^b^ n = 18	Last AB CF^b^ n = 18
**Cotrimoxazole**^a^	69.9%	88.5%	51.0%	<0.001	74.4%	72.2%	55.6%
**Piperacillin – tazobactam**^a^	96.5%	97.5%	95.4%	0.2	97.4%	94.4%	88.9%
**Meropenem**^a^**^,^**^e^	81.9%	92.6%	71.1%	<0.001	92.3%	88.9%	72.2%
**Imipenem**^c^	87.6%	80.7%	94.6%	<0.001	92.3%	88.9%	94.4%
**Ceftazidime**^b^	72.6%	91.8%	53.1%	<0.001	84.6%	66.7%	61.1%
**Ciprofloxacin**^b^	5.4%	6.2%	4.6%	0.55	20.5%	11.1%	0.0%

First AB CF = first isolate of the study period in pwCF (one strain per species and per patient).

CF: Cystic Fibrosis; NCF: non Cystic Fibrosis.

Last AB CF = last isolate of the study period in pwCF(one strain per species and per patient).

CF vs NCF p-value: test result comparing the *rates* of susceptible strains in patients with CF and those without CF.

For all pwCF.

For pwCF for whom at least two strains of the same species and same pulsotype were isolated over the period.

a According to EUCAST 2024 « *Achromobacter xylosoxidans* » disk diffusion method.

b According to EUCAST 2019 « Pseudomonas aeruginosa » disk diffusion method.

c According to Harris et al., 2025 (proposition for CLSI 2026).

d One patient with 3 species and another with 2 species.

e Percentage taking into account the ‘susceptible’ and ‘Susceptible at increased exposure’ categories.

Piperacillin-tazobactam remained the most active antibiotic whatever the context (in pwCF or in pw/oCF). However, 6/36 (16.7%) of pwCF had at least one resistant strain during the study period (Table S2).

In longitudinal isolates from pwCF, we observed a general trend toward increased resistance over time, with a significant difference when the test was performed overall (i.e., including all antibiotics to perform the test) (Table 4 and Table S3).

Higher resistance rates to meropenem, cotrimoxazole, and ceftazidime were observed in pwCF compared to pw/oCF with a significant difference (p < 0.001) but susceptibility to imipenem in pwCF was higher (p < 0.001).

No acquired resistance to aminoglycosides was detected among the isolates belonging to the intrinsically susceptible species (*A. mucicolens, A. kestersii, A. animicus* and *A. spanius*) (absence of the RND-efflux system AxyXY-OprZ).

For ciprofloxacin, ceftazidime and imipenem, *A. xylosoxidans* strains were significantly more resistant than the other species (p < 0.05) (Table S4). In pw/oCF, we noted that all the strains of *A. insuavis* studied were susceptible to piperacillin-tazobactam, imipenem and ceftazidime.

## 4 Discussion

*Achromobacter* are opportunistic multi-resistant pathogens mostly reported among pwCF but increasingly outside this context, with very few epidemiological data available to date. This is why we conducted this retrospective study, which aimed to describe exhaustively all the samples in which *Achromobacter* was detected and reported to clinicians in our hospital over a 6-year- period, including both pwCF or pw/oCF. One of the strengths of our study is the accurate *Achromobacter* species identification, thanks to our local MALDI-TOF MS database used since 2021 [8,25] and completed for some isolates by *nrdA*-sequencing when necessary. The limitation of our local database concerns the identification of the species for which only few isolates have been used for its construction and the species not included, such as *A. aestuarii*, *A. aloeverae*, and *A. seleniivolatilans*, or other species not yet described. Also, these novel species are absent from the Pubmlst database and therefore cannot currently be identified through analysis of the *nrdA* gene either.

Despite the monocentric nature of this study, a large collection of strains has been included (n = 651) from a great number of patients (n = 274). Interestingly during 2018-2023, when excluding samples from pwCF and screening for MDRGN, *Achromobacter* spp. has been isolated from an average of 38 patients each year in our hospital. Another similar study conducted in Germany in a tertiary care-hospital of 1300-beds included 314 clinical and 130 screening samples from distinct patients during 18 years [23]. However, unlike in our study, Neidhofer's study focused on all isolates labelled biochemically or by MALDI-TOF-MS as *A. xylosoxidans* but no accurate method of identification of the strains to the species-level has been used*,* preventing reporting the data by species. Similary, very recently Bayless conducted a 10-year retrospective study in three tertiary care centers in United States (US), including 1598 isolates not identified to the species level with accurate methods [24]. Furthermore, these two studies reported data on respiratory samples without distinguishing between pwCF and pw/oCF.

Regarding the distribution of sample types outside the context of CF, *Achromobacter* was mainly found in respiratory samples, which is consistent with Bayless's findings, followed by ear samples, blood cultures and urine samples. In Neidhofer's study, respiratory samples accounted for only 24% of clinical samples whereas urinary tract positive samples were more frequently detected (22%) than in our study. In our centre, positive respiratory samples mainly originated from patients in respiratory or intensive care units suffering from chronic respiratory diseases (COPD or bronchiectasis) or ventilator-associated pneumonias (VAP), already described as underlying diseases [29]. *Achromobacter* was also frequently isolated from ear samples. This observation was already made during 2010-2015 in our hospital [5] but at that time we did not detect any cases of otitis externa. Interestingly *Achromobacter* was regularly detected in bone infections mostly in case of diabetic foot infections (polymicrobial samples) unlike what is reported [4,23]. No cases of central nervous system infection or endocarditis were detected, confirming their rare occurrence [1,24].

With regard to pwCF, the global prevalence of *Achromobacter* colonization during the study period was of 20.3 % in our CF Center (36/177 patients), with a chronic colonization rate of 4.5 % (8/177), which is consistent with former studies conducted in our center (colonization of 26.6% of patients and chronic colonization of 6.9% of patients) [30]. In a study conducted in Madrid, the colonization rate was 11% and the chronic colonization rate was 5.8% [31]. In our study, around a quarter of patients carrying *Achromobacter* were considered chronically colonized. This rate increases if the *A. xylosoxidans* species is considered. Indeed, about half patients from whom *A. xylosoxidans* was isolated were chronically colonized. These findings were already observed in our previous studies in our center [30], and in our multicentric study [25] emphasizing once again the ability of this species to persist in pwCF respiratory tract. It is noteworthy however that the number of colonized patients dropped sharply during the period from 13 in 2021 (8.7%) to only 3 patients in 2022 (2.5%). One explanation is probably the introduction in recent years of CF transmembrane conductance regulator modulator therapies such as ivacaftor/tezacaftor/elexacaftor (widely used in our center since 2020). These results are consistent with other studies reporting a decrease in infections, prevalence, and bacterial density in the lungs with regard to *Achromobacter* [32,33].

A large diversity of species was detected, the most frequent species being *A. xylosoxidans,* whether in the context of CF or not, as already described in the literature [[3], [4], [5],^11^,^28^]. Furthermore, we support the hypothesis mentioned previously that the distribution of certain species, such as *A. ruhlandii* and *A. dolens*, varies between countries, perhaps sometimes because of the circulation of epidemic clones, such as the *A. ruhlandii* Danish epidemic strain in Denmark [11]. We did not detect these species, or detected very few of them, as in previous studies conducted in France and Italy [5,11,25]. In contrast, these species are observed much more frequently in studies conducted in the United States, Argentina, in the United Kingdom and Denmark (with the exception of *A. dolens* in this country) [11]. Interestingly, outside CF, the species *A. xylosoxidans* was significantly more detected in respiratory samples *versus* non respiratory samples, suggesting maybe a tropism of this species for respiratory tract.

In this study, we also sought to describe the context in which the strains were isolated. As *Achromobacter* are opportunistic pathogens, the isolation of these bacteria in samples is not systematically associated with infection and the interpretation and therapeutic management remains challenging, particularly in cases of polymicrobial cultures. In addition, the improvement of bacterial identification techniques in recent years (like MALDI-TOF-MS) might have enhanced the detection of these bacteria among polymicrobial samples. For interpretation purposes, the type of sample should be taken into account. For example, in case of foot diabetic the detection of *Achromobacter* in a bone sample should be considered, whereas it is difficult to conclude in case of superficial sample. Also, it is difficult to conclude on the pathogenicity of *Achromobacter* when associated with *P. aeruginosa* in respiratory samples [1]. In pwCF it has been shown that the presence of *Achromobacter* is a risk factor for disease progression and should be taken into account [3,31,34]. Outside CF, very few studies have examined the mono or polymicrobial nature of samples in which *Achromobacter* was isolated.

In our study, more than 80% of isolates were derived from multi-microbial samples (frequently associated with *P. aeruginosa*). Neidhofer reported that urine and wound samples were most of the time polymicrobial [23]. In Bayless's study, half of the blood stream infections (BSI) were polymicrobial [24].

Therefore, we focused on the 47 patients in whom a monomicrobial context was identified*.* Almost all of these patients (except for patients with *Achromobacter*-positive ear cultures and one patient with *Achromobacter*-positive respiratory cultures) harboured risk factors already described [1,15,29,35,36]. *A. xylosoxidans* was the main involved species.

With regard to the epidemiology of resistance, most studies focused on the context of CF, which is a very specific population receiving iterative antibiotic therapies [37] and our data did indeed indicate an increase in resistance over time. Some studies report resistant rates outside this context, but without correct species identification [23,24], and finally several studies report resistance rates in collection of strains not reflecting the epidemiology in a single center [4,28]. Because of the retrospective design of this study, we could not determine MICs, but we decided to reinterpret the antibiotic inhibition diameters according to the recommendations that appeared to be the most recent or accurate for this bacterial genus, whether European or American. As previously reported, piperacillin-tazobactam was the most active antibiotic, followed by imipenem, meropenem, ceftazidime and cotrimoxazole. The description of resistance varies between countries and populations studied [1], but piperacillin-tazobactam is the molecule most often described as the most effective against *Achromobacter* [4,37]. Is has to be noted however that *A. ruhlandii* species were not detected in this study, although described with poor sensibility to piperacillin-tazobactam and meropenem among pwCF [37,38]. When comparing the species *A. xylosoxidans* to non*-xylosoxidans Achromobacter* species, we observed that *A. xylosoxidans* isolates were significantly more resistant to imipenem, ceftazidime and ciprofloxacin, than non*-xylosoxidans Achromobacter* species isolates. Notably, no strain of *A. xylosoxidans* was found susceptible to ciprofloxacin unlike other species. This is consistent with most studies focusing on *A. xylosoxidans strico sensu*. In Olbrecht's study [37], among 175 isolates from pwCF, none of the strains harbored ciprofloxacin MIC <1 mg/l. This could suggest that *A xylosoxidans* is intrinsically poorly susceptible to ciprofloxacin. This could be explained by the fact that intrinsic RND efflux systems are capable of extruding fluoroquinolones in this species: AxyABM, AxyXY-OprZ, AxyEF-OprN, AxySUV [17,20,21,39]. Levofloxacin (not tested here) may be of interest, as several studies have classified more strains as susceptible compared to ciprofloxacin [24]. As expected, we observed significant higher resistance rates for meropenem, cotrimoxazole and ceftazidime in isolates from pwCF compared to pw/oCF. Surprisingly, imipenem resistance rate was significantly lower in isolates from pwCF, compared with other patients, resulting in higher activity of imipenem *versus* meropenem in this population. This particular phenotype, already reported in our CF center and by some authors [27,40], might be explained by overexpression of efflux systems: for example, AxyABM, which are able to extrude carbapenems, but preferentially meropenem rather than imipenem [20]. This efflux overexpression would be more frequently encountered among isolates of pwCF because of the iterative antibiotic therapies. Unfortunately, CASFM-EUCAST does not propose critical diameters for imipenem but the future publication in the next CLSI will be of real interest in this population [28].

In summary, it is important to consider the species and the clinical context when selecting the most appropriate treatment. While species like *A. xylosoxidans* or *A. insuavis* harbor intrinsic aminoglycosides resistance due to the AxyXY-OprZ efflux system [41], the use of these antibiotics should be considered for species lacking this system (e.g., *A. mucicolens, A. animicus*), as no acquired resistance was detected in our study. Similarly, ciprofloxacin should be avoided to treat *A. xylosoxidans* infections, whereas it could be used for other species. Finally, our data also suggest that meropenem should be preferred over imipenem for *A. xylosoxidans* in pw/oCF while imipenem could be useful to treat pwCF.

## 5 Conclusions

To conclude, this work enabled us to establish an overview of the involvement of the genus *Achromobacter* in the infection and/or colonization of patients in our hospital over a six-year period. This is one of the first studies to report data on the context of *Achromobacter* detection, using accurate methods of species identification and allowing comparison of resistant rates both between species and according to the CF status of the patients. These data highlight the influence of the species and the patient's pathology on antibiotic resistance rates. These factors should be taken into account when managing antibiotic therapies or, in the long term, when developing treatment recommendations.

## Data availability statement

Given the sensitive nature of the data collected for this study, patients participating in the survey were assured that the raw data would remain confidential and would not be disclosed. Data not available/The data used are confidential.

## Acknowledgements and financial support

This work was funded by an internal call for projects from the Dijon Bourgogne University Hospital (AOI 2024). The funders had no role in study design, data collection and interpretation, or the decision to submit the work for publication.

## CRediT authorship contribution statement

**Léa Cultrera:** Conceptualization, Data curation, Formal analysis, Investigation, Methodology, Software, Validation, Visualization, Writing – original draft, Writing – review & editing. **Caroline Demeule:** Formal analysis, Investigation, Visualization, Writing – original draft, Writing – review & editing. **Catherine Neuwirth:** Conceptualization, Funding acquisition, Methodology, Resources, Validation, Writing – original draft, Writing – review & editing. **Anne Claire Saussier:** Investigation. **Véronique Varin:** Investigation. **Lucie Amoureux:** Conceptualization, Formal analysis, Funding acquisition, Investigation, Methodology, Project administration, Resources, Supervision, Validation, Visualization, Writing – original draft, Writing – review & editing. **Arnaud Magallon:** Conceptualization, Data curation, Formal analysis, Funding acquisition, Investigation, Methodology, Project administration, Resources, Software, Supervision, Validation, Visualization, Writing – original draft, Writing – review & editing.

## Declaration of competing interest

The authors declare that they have no known competing financial interests or personal relationships that could have appeared to influence the work reported in this paper.

## Abbreviations used:

pwCF: patients with cystic fibrosis
MLST: MultiLocus Sequence Typing
MALDI-TOF-MS: matrix-assisted-laser-desorption/ionization-time-of-flight mass spectrometry
pw/oCF: patients without CF
CLSI: Clinical and Laboratory Standards Institute
MDRGN: multidrug resistant Gram negative bacteria
COPD: chronic obstructive pulmonary disease
