# Supplementary material for: Epidemiology of Achromobacter in a French hospital over six years: sample types, species, and antibiotic resistance profiles
Source: New Microbes New Infect. 2026 May 30;72:101777. doi: 10.1016/j.nmni.2026.101777 (PMC13263740; doi:10.1016/j.nmni.2026.101777)
Supplement: Multimedia component 1 [file mmc1.docx]

|  | **EUCAST 2024**  ***Achromobacter xylosoxidans*** | | | **EUCAST 2019**  ***Pseudomonas aeruginosa*** | | **Harris *et al* 2025**  ***Achromobacter spp*** | | |
| --- | --- | --- | --- | --- | --- | --- | --- | --- |
|  | S ≥ | SIE | R < | S ≥ | R < | S ≥ | I | R < |
| **Cotrimoxazole** | 26 | - | 26 |  | |  | | |
| **Piperacillin - tazobactam** | 26 | - | 26 |  | |  | | |
| **Meropenem** | 26 | 20-25 | 20 |  | |  | | |
| **Imipenem** |  | | |  | | 24 | 18-23 | 18 |
| **Ceftazidime** |  | | | 17 | 17 |  | | |
| **Ciprofloxacin** |  | | | 26 | 26 |  | | |

Table S1: Disk breakpoints, in mm, used for zone diameters interpretation:

*S = sensitive*

*R = resistant*

*SIE = Susceptible at increased exposure*

*I = intermediate*

*- = no recommendations*

Table S2: Number of patients with CF with at least one non susceptible strain over the period.

| **Antibiotic** | Number of patients  (n=36) |
| --- | --- |
| **Cotrimoxazole** | 17  **47.2%** |
| **Piperacillin – tazobactam** | 6  **16.7%** |
| **Meropenem** | 9  **25.0%** |
| **Imipenem** | 7  **19.4%** |
| **Ceftazidime** | 11  **30.6%** |
| **Ciprofloxacin** | 32  **88.9%** |

Table S3: Antibiogram results for strains isolated from pwCF who had at least two *Achromobacter* strains during the study period (n=18)

| **Patient** | **Espèce** | **Piperacillin-tazobactam^1^** | | **Meropenem^1, #^** | | **Cotrimoxazole^1^** | | **Imipeneme^3^** | | **Ceftazidime^2^** | | **Ciprofloxacin^2^** | |
| --- | --- | --- | --- | --- | --- | --- | --- | --- | --- | --- | --- | --- | --- |
|  |  | **First AB** | **Last AB** | **First AB** | **Last AB** | **First AB** | **Last AB** | **First AB** | **Last AB** | **First AB** | **Last AB** | **First AB** | **Last AB** |
| **1** | *A. xylosoxidans* | S | S | S | R | S | S | S | S | R | R | R | R |
| **2** | *A. xylosoxidans* | S | S | S | S | S | S | S | S | S | R | R | R |
| **3** | *A. xylosoxidans* | S | S | S | S | S | R | S | S | S | S | R | R |
| **4** | *A.* other | S | S | S | S | S | S | S | S | S | S | R | R |
| **5** | *A. xylosoxidans* | S | S | S | S | S | S | I | S | S | S | R | R |
| **6** | *A. insuavis* | S | S | S | S | S | S | S | S | S | S | S | R |
| **7** | *A. xylosoxidans* | R | R | SIE | S | R | R | S | S | R | R | R | R |
| **8** | *A. insuavis* | S | R | S | R | S | R | S | S | S | R | R | R |
| **9** | *A. xylosoxidans* | S | S | R | R | R | R | S | S | R | R | R | R |
| **10** | *A. xylosoxidans* | S | S | SIE | R | R | R | S | S | R | R | R | R |
| **11** | *A. xylosoxidans* | S | S | R | R | R | R | S | S | R | R | R | R |
| **12** | *A. xylosoxidans* | S | S | S | S | S | S | S | S | S | S | R | R |
| **13** | *A. insolitus* | S | S | S | S | S | R | S | S | S | S | R | R |
| **14** | *A. mucicolens* | S | S | S | S | S | R | S | S | S | S | S | R |
| **15** | *A. xylosoxidans* | S | S | S | S | S | S | S | S | S | S | R | R |
| **16** | *A. xylosoxidans* | S | S | S | S | R | S | I | S | R | S | R | R |
| **17** | *A. xylosoxidans* | S | S | SIE | SIE | S | S | S | S | S | S | R | R |
| **18** | *A. xylosoxidans* | S | S | S | S | S | S | S | I | S | S | R | R |

*S (green)= sensitive ; SIE (light green) = Susceptible at increased exposure; I (orange)= intermediate ; R (red) = resistant*

*First AB = Therapeutic classification based on the antibiogram of the first strain isolated during the study period*

*Last AB = Therapeutic classification based on the antibiogram of the last strain isolated during the study period*

*^1^: According to EUCAST 2024 « Achromobacter xylosoxidans » disk diffusion method*

*^2 :^ According to EUCAST 2019 « Pseudomonas aeruginosa » disk diffusion method*

*^3^: According to Harris et al. 2025 (proposition for CLSI 2026)*

*The exact McNemar test is used to compare “first AB” VS “last AB” values, both on a molecule-by-molecule basis and overall (considering S and SIE to have the same value, and similarly for R and I).*

Table S4: Antibiotic susceptibility of *Achromobacter* strains isolated from patients without cystic fibrosis, by species, (susceptible (S) or susceptible at increased exposure (SIE))

| Antibiotic | species | % of S or SIE | *p*-value |
| --- | --- | --- | --- |
| Cotrimoxazole^1^ | ***A. xylosoxidans* (n=179)** | 87.7% | 0.39 |
|  | ***non-xylosoxidans Achromobacter species* (n=60)** | 91.7% |  |
|  | ***A. insuavis* (n=22)** | 95.5% |  |
| Piperacillin– tazobactam^1^ | ***A. xylosoxidans* (n=179)** | 97.2% | 0.63 |
|  | ***non-xylosoxidans Achromobacter species* (n=60)** | 98.3% |  |
|  | ***A. insuavis* (n=22)** | 100% |  |
| Meropenem^1^ | ***A. xylosoxidans* (n=179)** | 91.1% | 0.15 |
|  | ***non-xylosoxidans Achromobacter species* (n=60)** | 96.7% |  |
|  | ***A. insuavis* (n=22)** | 95.5% |  |
| Imipenem^3^ | ***A. xylosoxidans* (n=179)** | 75.4% | < 0.001 |
|  | ***non-xylosoxidans Achromobacter species* (n=60)** | 98.3% |  |
|  | ***A. insuavis* (n=22)** | 100% |  |
| Ceftazidime^2^ | ***A. xylosoxidans* (n=179)** | 89.4% | 0.032 |
|  | ***non-xylosoxidans Achromobacter species* (n=60)** | 98.3% |  |
|  | ***A. insuavis* (n=22)** | 100% |  |
| Ciprofloxacin^2^ | ***A. xylosoxidans* (n=179)** | 0 % | < 0.001 |
|  | ***non-xylosoxidans Achromobacter species* (n=60)** | 23.3% |  |
|  | ***A. insuavis* (n=22)** | 27.3% |  |

*Proportions established on 239 strains from patients without cystic fibrosis.*

*SIE = susceptible at increased exposure*

*^1:^ According to CASFM 2024 « Achromobacter xylosoxidans »*

*^2:^ According to CASFM 2019 « Pseudomonas aeruginosa »*

*^3^: According to Harris et al. 2025 (proposition for CLSI 2026)*

*p-value: test result comparing the rates of susceptible strains in A. xylosoxidans and non-xylosoxidans Achromobacter species.*
